# Runx2 mediated Induction of Novel Targets ST2 and Runx3 Leads to Cooperative Regulation of Hypertrophic Differentiation in ATDC5 Chondrocytes

**DOI:** 10.1038/s41598-017-18044-z

**Published:** 2017-12-20

**Authors:** Ehsan Bonyadi Rad, Giuseppe Musumeci, Karin Pichler, Maryam Heidary, Marta Anna Szychlinska, Paola Castrogiovanni, Egon Marth, Christina Böhm, Sriveena Srinivasaiah, Gerhard Krönke, Annelie Weinberg, Ute Schäfer

**Affiliations:** 1 Department of Orthopedics and Trauma Surgery, Medical University Graz, Graz, Austria; 2 Department of Biomedical and Biotechnological Sciences, Human Anatomy and Histology Section, School of Medicine, University of Catania, Catania, Italy; 3 Department of Children and Adolescent Medicine, Pediatrics I, Medical University of Innsbruck, Innsbruck, Austria; 4 Division of Neonatology, Pediatric Intensive Care and Neuropediatrics, Department of Pediatrics, Medical University of Vienna, Vienna, Austria; 5 Translational Research Department, Institute Curie, Paris, France; 6 Institute of Hygiene, Microbiology and Environmental Medicine, Medical University Graz, Graz, Austria; 7 Friedrich-Alexander-University Erlangen-Nürnberg (FAU), Department of Internal Medicine 3 – Rheumatology and Immunology, Universitätsklinikum Erlangen, Erlangen, Germany; 8 Department of Neurosurgery, Medical University Graz, Graz, Austria

## Abstract

Knowledge concerning expression and function of Suppression of Tumorigenicity 2 (ST2) in chondrocytes is at present, limited. Analysis of murine growth plates and ATDC5 chondrocytes indicated peak expression of the ST2 transmembrane receptor (ST2L) and soluble (sST2) isoforms during the hypertrophic differentiation concomitant with the expression of the hypertrophic markers Collagen X (Col X), Runx2 and MMP-13. Gain- and loss-of-function experiments in ATDC5 and primary human growth plate chondrocytes (PHCs), confirmed regulation of ST2 by the key transcription factor Runx2, indicating ST2 to be a novel Runx2 target. ST2 knock-out mice (ST2−/−) exhibited noticeable hypertrophic zone (HZ) reduction in murine growth plates, accompanied by lower expression of Col X and Osteocalcin (OSC) compared to wild-type (WT) mice. Likewise, ST2 knockdown resulted in decreased Col X expression and downregulation of OSC and Vascular Endothelial Growth Factor (VEGF) in ATDC5 cells. The ST2 suppression was also associated with upregulation of the proliferative stage markers Sox9 and Collagen II (Col II), indicating ST2 to be a new regulator of ATDC5 chondrocyte differentiation. Runx3 was, furthermore, identified as a novel Runx2 target in chondrocytes. This study suggests that Runx2 mediates ST2 and Runx3 induction to cooperatively regulate hypertrophic differentiation of ATDC5 chondrocytes.

## Introduction

ST2 (also known as IL1RL1, T1, FIT-1 and DER4), a so-called primary response gene, was originally identified through its induction in murine fibroblasts by serum, growth factors or oncogenes (Ha-ras and v-mos)^1–4^. ST2 is a member of the Toll-like/IL-1 receptor superfamily and encodes two major isoforms, ST2L and sST2, as well as two other isoforms, ST2V and ST2LV, by alternative splicing^5–7^. The ST2 gene is regulated by distal and proximal promoters in a highly cell-type specific manner^8–11^.

Developing bone is one of the prominent sites of ST2 expression^12,13^. Initial studies of the expression pattern of ST2 in mandibular condyle, an ideal model for studying various types of skeletoblasts, revealed a temporal expression pattern of sST2. These results were supported by increased ST2 gene expression at early stages of differentiation during *in vitro* culture of the osteoblast-like cell lines MC3T3-E1 and KM-1K^12^. In another investigation, ST2L silencing in osteosarcoma cells cultured in a 3-dimensional collagen I matrix resulted in the suppression of the osteoblastogenesis markers collagen I, OSC and alkaline phosphatase expression^14^.

Runx2 is a key transcription factor in osteoblast differentiation and bone formation^15,16^ and also plays an important role in chondrocyte differentiation^17^. Runx2 is extensively expressed in pre-hypertrophic and hypertrophic chondrocytes and knockout (Runx2−/− mice) results in severely disturbed chondrocyte differentiation^17,18^. The growth plate, a cartilaginous tissue with a unique morphological structure, comprises an array of chondrocytes organized into resting, proliferative and hypertrophic zones^19,20^. Terminally differentiated hypertrophic chondrocytes eventually undergo degeneration and apoptosis^20–23^ and also have the capability to trans-differentiate to osteoblast cells according to recent evidence^24–26^. Longitudinal bone growth occurs through a process of endochondral ossification within the growth plate, which gradually converts avascular cartilage into highly vascularized bony tissue^27^.

The present study investigated the expression, regulation, and function of ST2 in chondrocytes which, to date, have not been addressed. Following an assessment of the pattern of ST2 expression in different murine tibia and femur growth plate zones, expression of ST2 splice variants was analyzed during sequential stages of ATDC5 differentiation. The key transcription factor Runx2 was shown to induce expression of ST2 isoforms in ATDC5 and PHCs. Analysis of ST2 knockout mice revealed perceptible hypertrophic zone size reduction associated with a decreased expression of Col X and OSC comparable to the *in vitro* effect of ST2 silencing in ATDC5 cells. We further identified Runx3 as a novel target of Runx2 in chondrocytes. Eventually, Runx2 associated enhancement of hypertrophic markers Col X, OSC, VEGF, and MMP-13 was decreased by ST2 and Runx3 knockdown thus indicative of cooperative regulation of ATDC5 hypertrophic differentiation driven by Runx2 mediated activation of the downstream targets ST2 and Runx3.

## Materials and Methods

### Cell culture

Murine chondrogenic cell line ATDC5 was obtained from European collection of cell cultures (ECACC, Salisbury, UK). ATDC5 cells were maintained in Dulbecco’s Modified Eagle’s Medium/Nutrient F-12 Ham media containing 5% (v/v) fetal bovine serum (PPA Pasching Austria), 10 μg/ml human transferrin (Sigma-Aldrich St. Louis, MO) and 3 × 10^−8^ M sodium selenite (Sigma Chemical, St Louis, MO, USA). To induce differentiation, maintenance media was supplemented with 10 µg/ml bovine insulin (Sigma-Aldrich St. Louis, MO) and 37,5 µg/ml ascorbic acid (Wako pure chemical industries) according to previous study^28^. ATDC5 cells were incubated in a humidified atmosphere at 37 °C, 5% CO2. The medium was changed every other day.

### Isolation and culture of primary human growth plate chondrocytes (PHCs)

PHCs were isolated from growth plate of the supernumerary digits of five different donors with polydactylism (3 males and 2 females) with ages ranging from 0.25 to 6 years at the time of surgery. Informed consent was obtained from the parents or any legal representative. The PHCs were obtained by digesting the growth plate tissue in 2 mg/ml collagenase B (150 units/mg collagenase B, Worthington Biochemical Corp., Lakewood, NJ, USA) in a shaking water bath at 37.5 °C overnight. The isolated cell suspensions filtered through two layers of nylon grid with 40 μm mesh size and then expanded in growth media (Dulbecco’s modified Eagle’s medium/nutrient mixture F-12 supplemented with 5% FBS and 2 mM glutamine). Cells were then incubated at 37 °C, 5% CO2 in a humidified atmosphere. PHCs in passage one or two were used in all experiments. Ethical approval for this study was obtained from the Ethics Committee of the Medical University of Graz and related experiments were performed in accordance with relevant guidelines and regulations.

### Quantitative Reverse Transcription Polymerase Chain Reaction (qPCR)

The qPCR was performed according to the previous study^29^. As per manufacturer’s instructions total RNA was extracted from ATDC5 chondrocytes, MC3T3-E1 osteoblasts and primary human growth plate chondrocytes using RNeasy Mini Kit (Qiagen, Hilden, Germany). Potential DNA contamination was digested with DNase I (Qiagen, Hilden, Germany) during RNA purification according to manufacturer’s protocol. The cDNA was prepared from 2 µg of total RNA using the First Strand cDNA Synthesis Kit (Fermentas, St. Leon-Rot, Germany). The SYBR Green Master Mix from Invitrogen was used for PCR amplification and PCR products were detected by AB7900 HT TaqMan PCR system (Applied Biosystems). In differentiation experiments, 18 S were used as internal control. Relative expression was calculated according to the 2^−ΔΔCt^ method.

### Reverse Transcription PCR (RT-PCR) and promoter usage analysis using RT-PCR

Total RNA isolation and cDNA synthesis are as stated before. Prepared cDNAs were then used in subsequent PCR reactions using phusion high-fidelity DNA polymerase (Thermo scientific- Fisher Scientific - Austria GmbH). Following primers were used to examine the expression of murine ST2L and sST2 from distal and proximal promoters:

Murine distal first exon (E1a) forward primer was 5′GAATAAAGATGGCTAGGACCTCTGG3′, proximal first exon (E1b) forward primer was 5′AATGAGACGAAGGAGCGCCAAGTAG3′ and the reverse primers were 5′GCCACTCAACGGAGCCGCAA3′ for ST2L and 5′ACCAATACCAATGTCCCTTGTAGTCGG3′ for sST2^10^. Promoter primers used for human samples were: exon1a (E1a) forward primer: 5′GAGAAATTGGCTTTCTGAGTTGTGAAACTGTGGGC3′; exon1b (E1b) forward primer: 5′TCACTGACTCCAAGTTCATCCCCTCTGTCTTTCAG3′ and reverse primers were 5′TCAAACTCAGATGCCTTTGCACATCACAGCAGGCA3′ for ST2L and 5′CTCTTGGAACCACACTCCATTCTGCTTACACTTGC3′ for sST2 as reported by Iwahana *et al*.^8^. All PCR fragments were observed on 1% ethidium bromide stained agarose gels. Table 1 illustrates the qPCR and RT-PCR primers used in this study.

**Table 1:** List of qPCR and RT-PCR primers used in this study.

Primer sequences, qPCR	Reference Supplementary
Murine ST2L: F5′GCCCGACGTTCTTGAAAATA3′, R5′ATCTCCTGCTCGTAGGCAAA3′	This Study
Murine ST2 (Total): F5′CCAGCCAGAGTGGAAGACTC3′, R5′CAGGTCAATTGTTGGACACG3′	This Study
Murine Runx2: F5′TGTTCTCTGATCGCCTCAGTG3′, R5′CCTGGGATCTGTAATCTGACTCT3′	(1)
Murine Collagen II: F5′ACCTTGGACGCCATGAAA3′, R5′GTGGAAGTAGACGGAGGAA3′	(2)
Murine Collagen X: F5′TCTGGGATGCCGCTTGT3′, R5′CGTAGGCGTGCCGTTCTT3′	(2)
Murine MMP-13: F5′TGATGAAACCTGGACAAGCA3′, R5′CCTGGGTCCTTGGAGTGAT3′	(2)
Murine OSC: F5′AAGCAGGAGGGCAATAAGGT3′, R5′TAGGCGGTCTTCAAGCCATA3′	(3)
Murine Runx3: F5′ACCACGAGCCACTTCAGCAG3′, R5′CGATGGTGTGGCGCTGTA3′	(4)
Murine VEGF: F5′ CCCGGACGGGCCTCCGAAAC3′,R5′ AGCCTGGGACCACTTGGCATGG3′	(5)
Human Runx3: F5′CAGAAGCTGGAGGACCAGAC′, R′5GTCGGAGAATGGGTTCAGTT3′	(6)
Human sST2: F5′GGCTTGAGAAGGCACACCGT3′, R5′GGAGTGGGGGAGGACGAAC3′	(7)
Human Aggrecan: F5′ GCGAGTTGTCATGGTCTGAA3′, R5′TTCTTGGAGAAGGGAGTCCA3′	(8)
Human Collagen II: F5′CGGCTTCCACACATCCTTAT3′, R5′CTGTCCTTCGGTGTCAGGG3′	(8)
Human Collagen X: F5′GTGGACCAGGAGTACCTTGC3′, R5′CATAAAAGGCCCACTACCCA3′	(8)
Murine 18 S: F5′CGCCGCTAGAGGTGAAATTC3′, R5′TTGGCAAATGCTTTCGCTC3′	(9)
Murine GAPDH: F5′TGACCACAGTCCATGCCATC3′, R5′GACGGACACATTGGGGGTAG3′	(10)
Human GAPDH: F5′GAAGGTGAAGGTCGGAGTC3′, R5′GAAGATGGTGATGGGATTTC3′	(10)
**Primer sequences, RT-PCR**	
Murine ST2L: F5′TGCGTACATCATTTACCCTCGGGTC3′, R5′GCCACTCAACGGAGCCGCAA3′	(11)
Murine sST2: F5′AGCTGTGCACTGCATCCGTTTTC3′, R5′ACCAATACCAATGTCCCTTGTAGTCGG3′	(11)
Murine GAPDH: F5′GAAGGGCATCTTGGGCTACAC3′, R5′GCAGCGAACTTTATTGATGGTATT3′	(12)
Human ST2 forward Primer (common for both ST2L and sST2): 5′AGGCTTTTCTCTGTTTCCAGTAATCGG3′	(13)
Human ST2L Reverse Primer: 5′GGCCTCAATCCAGAACATTTTTAGGATGATAAC3′	(13)
Human sST2 Reverse Primer: 5′CAGTGACACAGAGGGAGTTCATAAAGTTAGA3′	(13)
Human GAPDH: F5′GAAGGTGAAGGTCGGAGTC3′, R5′GAAGATGGTGATGGGATTTC3′	(14)
Human Runx2 (PPH01897C)	Qiagen

### Gene silencing and overexpression experiments

Gene silencing and overexpression experiments were performed according to the previous study^29^. Two sets of siRNAs against Runx2 and ST2 were purchased from Dharmacon (Thermo Scientific) and Santa Cruz and siRNA targeting Runx3 was bought from Dharmacon (Thermo Scientific). Control siRNAs were obtained from Qiagen, Vienna, Austria. Gene silencing (150–200 pmol siRNA) analysis was performed 48 to 72 hrs post transfection. For overexpression experiments, cDNA clones encoding murine ST2L (NM_001025602), murine sST2 (NM_010743), murine (NM-001145920) and human (NM-001024630) Runx2 were purchased from Origene. In stable transfection experiments, cells were transfected with 3 µg of Runx2 cDNA vector or empty vector plasmids. 24 hrs post transfection cells were transferred to 25 cm flasks and selected with 400 µg/ml G418 antibiotics for the period of one to two weeks. In transient Runx2 transfection experiments (PHCs), gene expression analysis was performed 24 to 48 hrs post-transfection. All transfections were performed using Lipofectamine 2000 reagent (Invitrogen) according to the manufacturer’s protocol.

### Immunoblotting

Immunoblotting was performed according to the previous study^29,30^. After whole cell lysate preparation with RIPA buffer (Sigma-Aldrich St. Louis, MO), Bradford Protein Assay (BioRad, Hercules, CA) was used for measurement of protein concentration. 10 μg samples per lane were loaded on 10% SDS-polyacrylamide gels and then transferred to the polyvinylidene difluoride (PVDF) membranes (Millipore, Billerica MA). To avoid stripping, the membranes were cut and then probed with different primary antibodies. After overnight incubation with primary antibodies at 4 °C, membranes were incubated with respective peroxidase conjugated secondary antibodies. Proteins of interests were subsequently visualized using chemiluminescence (ECL) reagents (Amersham) and exposed to X-ray films (Kodak). Anti-DDK tag antibody was purchased from Origene, anti-Runx2 (D1L7F) and Runx3 (D6E2) antibodies were from Cell Signaling, anti-murine ST2 antibody (AP30829PU) was from Acris and antibody for detecting human ST2 (C-20) and β-actin (N21) were from Santa Cruz. All protein quantifications were normalized to their respective β-actin expression levels. Image J software (1.46r) was used for the quantification of the protein band intensities.

### Histochemistry and Immunohistochemistry (IHC)

Histochemistry and IHC experiments were performed according to the previous studies^31,32^. The mice were obtained from Akira Lab, Osaka University, Japan and were housed in the animal facility of the University of Erlangen–Nuremberg. Generation of the ST2−/− mice has been explained previously^33^. The mice were backcrossed 10 times on c57bl/6 background and genotyping of wild-type from homozygous ST2−/− mice was assessed by PCR using 5′TTGGCTTCTTTTAATAGGCCC3′ (wild-type), 5′CTATCAGGACATAGCGTTGGCTACC3′ (knockout) and 5′TGTTGAAGCCAAGAGCTTACC3′ (wild-type and knockout) primers before experimental investigations. Animal experiments were approved by the government of Mittelfranken and all methods were performed in accordance with the relevant guidelines and regulations. The specimens from male homozygous ST2−/− as well as WT mice (all c57bl/6 genetic background) at the age of 3 and 5 weeks (4 WT and 3 ST2−/− mice in each group) were washed by phosphate-buffered saline (PBS) and fixed in 10% buffered formalin for 24 hrs or 4% paraformaldehyde for 16 hrs at room temperature. Subsequently, the specimens were washed overnight, incubated in a decalcifying solution (14% EDTA, PH: 7,2) for 7–10 days, rinsed for 1 hr, dehydrated in graded ethanol, cleared in xylene and embedded in paraffin wax. A rotary microtome (Leica RM2235; Leica Microsystems, Wetzlar, Germany) was used to cut the sections with 3–4 µm thickness from paraffin blocks. The sections were then mounted on silane-coated slides (Dako, Glostrup, Denmark), and air-dried. To assess any structural alterations, the prepared slides were dewaxed using xylene, hydrated by graded ethanol, and stained with Hematoxylin and Eosin (H&E). For immunohistochemical analysis, the slides were first dewaxed using xylene and hydrated in graded ethanol and then incubated in 0.3% H_2_O_2_/methanol for 30 min to quench endogenous peroxidase activity. PBS was subsequently used for 20 min to wash the slides. 1-hour blocking was then performed by 5% bovine serum albumin (BSA; Sigma, Milan, Italy) in a humid chamber. The sections were subsequently incubated at 4 °C overnight with rabbit polyclonal anti-ST2 (ab25877; Abcam, Cambridge, UK), rabbit polyclonal anti-RUNX2 (ab23981; Abcam, Cambridge, UK), rabbit polyclonal anti-OSC (ab93876; Abcam, Cambridge, UK) and rabbit polyclonal anti-Col X (ab58632; Abcam, Cambridge, UK) all diluted 1:100 in PBS. Sections were then incubated with HRP-conjugated anti-rabbit secondary antibodies. The sections were incubated in a 0.1% 3,3′-diaminobenzidine (DAB) and 0.02% hydrogen peroxide solution (DAB substrate Chromogen System; Dako, Denmark) for 2 minutes for the observation of immunoreactions. The counterstaining of the sections were conducted lightly with Mayer’s hematoxylin (Histolab Products AB, Göteborg, Sweden) for 1 minute and mounted in GVA (Zymed Laboratories, San Francisco, CA, USA). The sections were visualized by an Axioplan Zeiss light microscope (Carl Zeiss, Oberkochen, Germany) and photographed with a digital camera (AxioCam MRc5, Carl Zeiss, Oberkochen, Germany).

### Evaluation of immunohistochemistry

Positive immunostaining was identified by light microscopy through detection of brown chromogen on the edge of the cell nucleus and/or distributed in the cytoplasm or plasma membrane. To analyze the protein reaction of primary antibodies (ST2, Runx2, OSC and Col X) positive and negative controls were used. The Intensity of Staining (IS) of ST2 (in trabecular bone and growth plate of femur and tibia) and Runx2 (in tibial growth plate) was tested and reported on a 0–4 scales as follow: no detectable staining = 0, weak staining = 1, moderate staining = 2, strong staining = 3, very strong staining = 4. The percentage of immunopositive cells was evaluated and reported through the five categories of Extent Score (ES): <5% (0); 5–30% (+); 31%–50% (++); 51–75% (+++), and >75% (++++). Counting was conducted under Zeiss Axioplan light microscope at ×200 magnification. The IHC analysis was assessed by 2 anatomical morphologists and one histologist. In case of interpretation dispute, the case was revised until a unanimous agreement was made. Morphometric analysis (length of different zones of the growth plate) were performed using a software for image acquisition and morphometric analysis (AxioVision Release 4.8.2 - SP2 Software, Carl Zeiss Microscopy GmbH, Jena, Germany) which measure size of femur and tibial proliferative and hypertrophic zones (PZ and HZ) by a semiautomatic analyses in which we determined the PZ and HZ in each slide. The calculated average size of each zone was then represented as a percentage relative to the full size of respective femur and tibia. The full size of each femur and tibia was measured by digital Vernier Caliper. Osteocalcin- and Collagen X-immunostained areas were calculated both in hypertrophic zone (red spot) and proliferative zone (green spot) of the growth plate using a software for image acquisition and densitometric analysis (AxioVision Release 4.8.2 - SP2 Software, Carl Zeiss Microscopy GmbH, Jena, Germany) which automatically quantifies the area of immunostaining in specific zones (HZ and PZ) determined by us, expressed as densitometric count (pixel^2^/unit area 1000 µm^2^) of positive, dark brown pixels. Zeiss Axioplan light microscope (Carl Zeiss, Oberkochen, Germany) equipped with a digital camera (AxioCam MRc5, Carl Zeiss, Oberkochen, Germany) was utilized to capture digital micrographs. For statistical analysis, comparisons between two means were evaluated by Mann Whitney test.

### Statistics

Data were presented as the mean ± SD. Statistical analysis between two means was assessed by Mann Whitney test whilst between means from more than two groups were evaluated by Kruskal–Wallis test with post Dunn’s test for multiple comparisons. Statistical analysis was performed using GraphPad Instat Biostatistics version 6.0 software (GraphPad Software, Inc. La Jolla, CA, USA).

### Data availability

All data generated during this study are included in this article and its supplementary information files.

## Results

### ST2 expression is strongly enhanced in hypertrophic chondrocytes in murine tibia and femur growth plates

**Figure 1:**
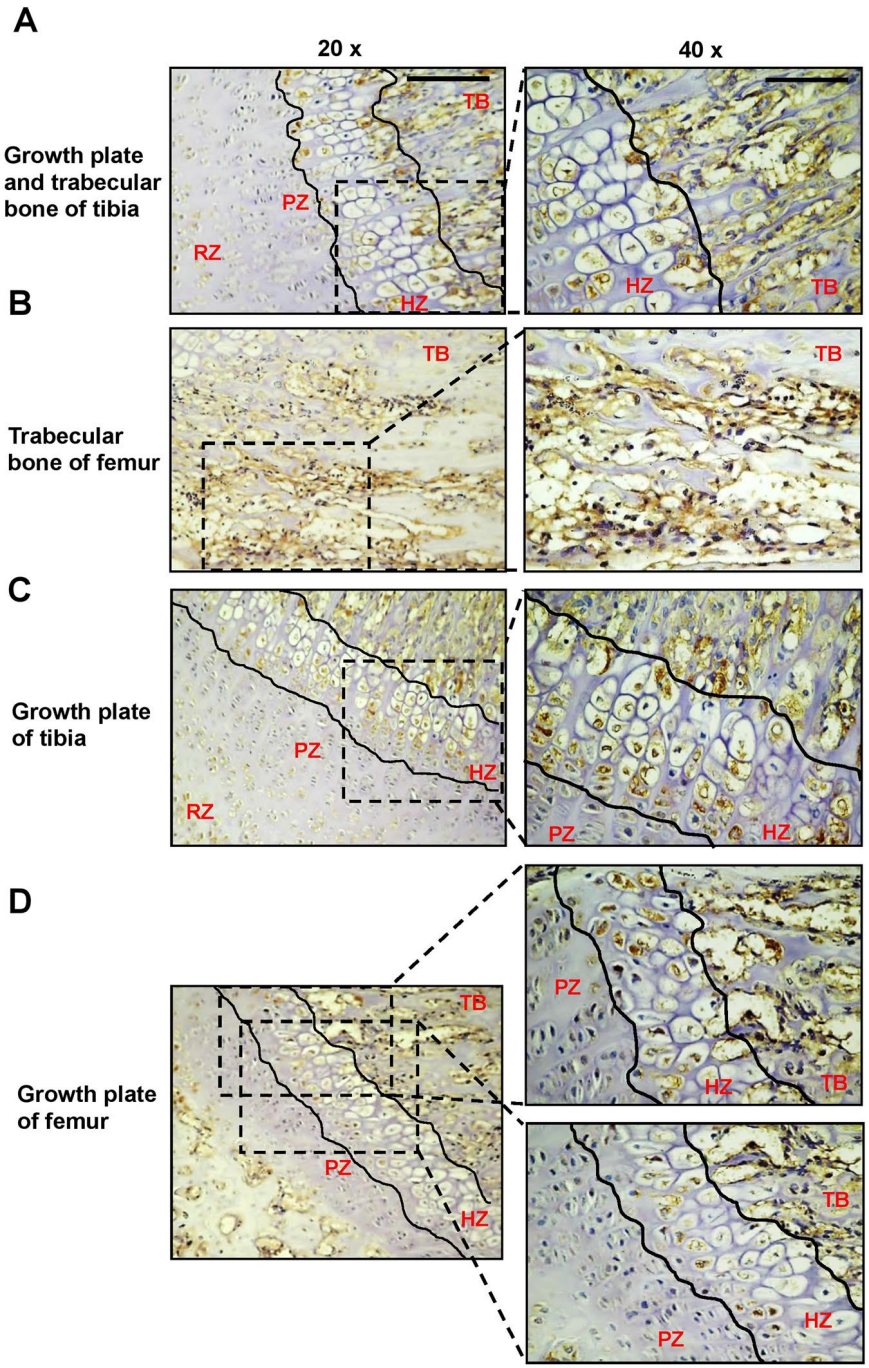
Expression pattern of ST2 in trabeculae and growth plate of murine tibia and femur. Immunohistochemical analysis of ST2 expression in the trabecular bone-growth plate of tibia (**A**) and trabecular bone of femur (**B**) and growth plate of tibia (**C**) and femur (**D**) from three week old mice. For each staining magnification of 20x with scale bars of 100 µm and 40x with scale bars of 50 µm are shown. RZ, resting zone, PZ, proliferative zone, HZ, hypertrophic zone and TB, trabecular bone.

Immunohistochemical (IHC) analysis revealed pronounced endogenous expression of the ST2 protein in both femur and tibial trabecular bone of three-week old mice (Supplemental Fig. 1, Fig. 1A,B, Table 2). Subsequent examination of ST2 expression in growth plate chondrocytes demonstrated differentiation-dependent variation in ST2 expression in both tibial and femur growth plate chondrocytes. Whereas IHC results showed low, or no ST2 expression in resting and proliferative zones, increased ST2 expression at the pre-hypertrophic stage and strong persistent expression at hypertrophic chondrocytes in both tibial and femur growth plates were observed (Fig. 1C,D, Table 2). These results, indicating upregulation of ST2 expression during late stages of chondrocyte differentiation, suggest a role for ST2 expression during chondrogenic hypertrophy.

**Table 2:** Evaluation of Intensity of staining (IS) and Extent Score (ES) of ST2 and Runx2 immunostaining in trabecular bone and growth plate of mice. (HZ, hypertrophic zone, PZ, proliferative zone and TB, trabecular bone).

	HZ	PZ	TB
FEMUR (ST2)	Strong immunostaining (ES = +++ ; IS = 3)	Weak immunostaining (ES = + ; IS = 1)	Very strong immunostaining (ES = ++++ ; IS = 4)
TIBIA (ST2)	Strong immunostaining (ES = +++ ; IS = 3)	Weak immunostaining (ES = + ; IS = 1)	Very strong immunostaining (ES = ++++ ; IS = 4)
TIBIA (Runx2)	Very strong immunostaining (ES = ++++; IS = 4)	Weak immunostaining (ES = + ; IS = 1)	Was not evaluated

### ST2 splice variants are robustly elevated in hypertrophic ATDC5

**Figure 2:**
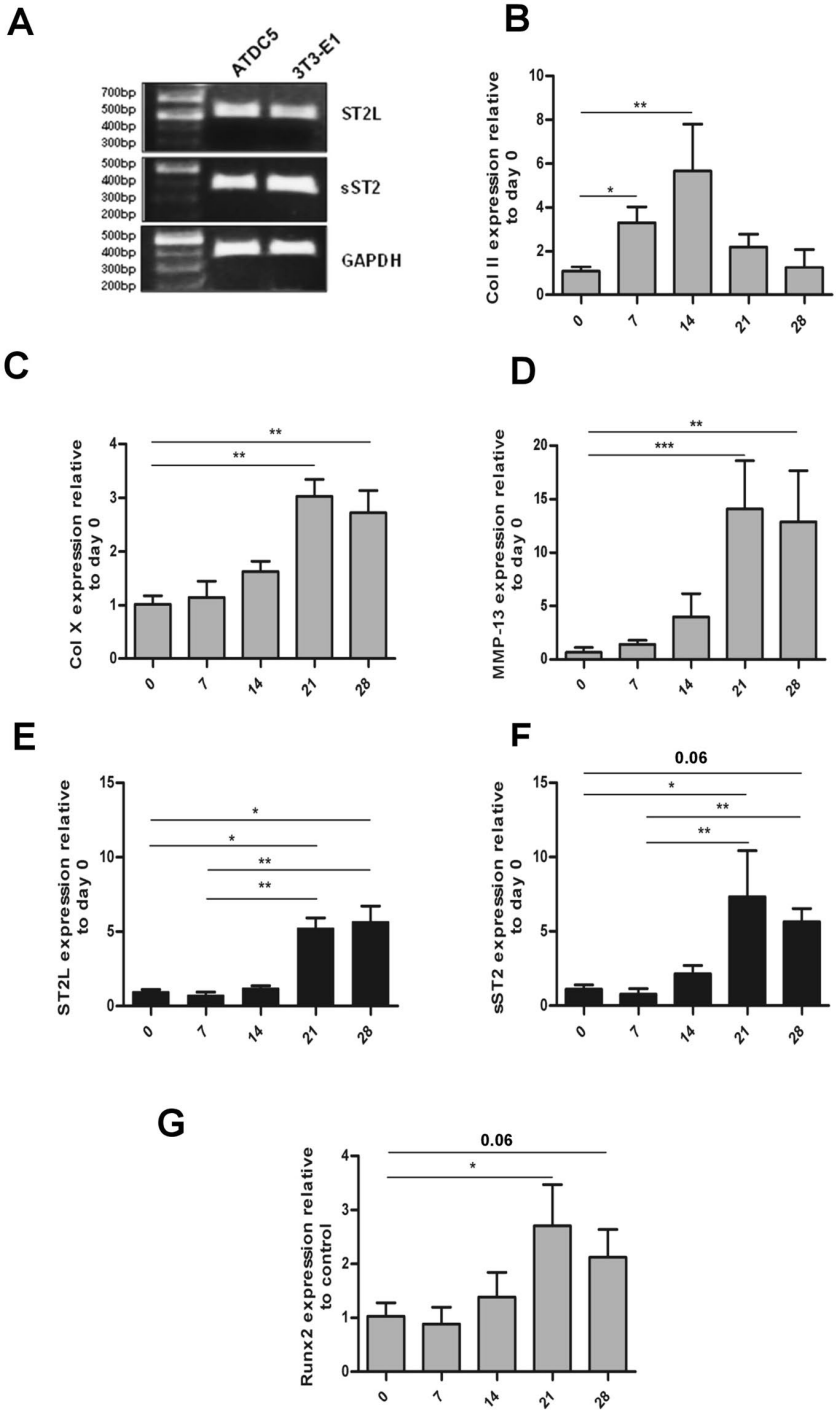
Expression of ST2L and sST2 splice variants during ATDC5 differentiation course. (**A**) RT-PCR examination of endogenous expression of transmembrane and soluble ST2 isoforms in ATDC5 and MC3T3-E1 cells (full gel is shown in Supplemental Fig. 2). PCR products with size 526 bp for murine ST2L and 360 bp for murine sST2 were observed on 1% agarose gels. Glyceraldehyde 3-phosphate dehydrogenase (GAPDH) was used as internal control. ATDC5 Cells were cultured in maintenance media for four days to reach confluence. Maintenance media were then changed to differentiation media and cells were incubated for the time course of 28 days. Total RNA isolated from cells at day 0, 7, 14, 21 and 28 was assessed by q-PCR for expression levels of Col II (**B**), Col X (**C**), MMP-13 (**D**), ST2L (**E**), sST2 (**F**) and Runx2 (**G**). The sST2 mRNA expression level is calculated using ST2/ST2L ratio. The expression level of each gene was normalized to 18 s rRNA and represented as the mean ± S.D. n = 5. Statistical significance are *P ≤ 0.05, **P < 0.01, ***P < 0.001.

In order to verify our IHC observations, expression of ST2L and sST2 isoforms was analyzed in the murine chondrogenic ATDC5 cell line, the pre-osteoblastic cell line MC3T3-E1 and during ATDC5 chondrocyte differentiation. Both splice variants were expressed in undifferentiated ATDC5 and MC3T3-E1 cell lines (Fig. 2A). The ATDC5 murine chondrogenic cell line has been shown to mimic *in vivo* sequential chondrocyte differentiation and cellular condensation in a multi-step differentiation process^34^. Expression of the ST2 isoforms was subsequently quantified during ATDC5 chondrogenic differentiation by qPCR during a period of twenty eight days in which cells undergo differentiation and induce expression of hypertrophic markers like Col X, MMP-13, OSC and Runx2 predominantly on days 21 to 28^35–37^. Chondrogenic differentiation was established by expression analysis of an early chondrogenic marker Col II and two hypertrophic markers, Col X and MMP-13 (Fig. 2B,D). The time-dependent expression pattern of the early and late phase chondrogenic markers indicated a progressive chondrogenic differentiation process. Both ST2L and sST2 expression levels were perceptibly, though not significantly reduced at day seven of this process. Strong, significant induction of both splice variants was observed during the hypertrophic stage from days 21 through 28, concomitant with enhanced expression of hypertrophic markers Col X and MMP-13. (Fig. 2E,F). These results are consistent with pronounced ST2 induction in hypertrophic chondrocytes in the murine growth plate (Fig. 1C,D), and suggest that ST2 likely plays a role in the late stages of chondrocyte differentiation. Runx2 has been previously shown to be involved in the regulation of chondrocyte maturation^17,18,38,39^. We accordingly observed simultaneous upregulation of Runx2 and ST2L/sST2 during the late stages of ATDC5 chondrogenic differentiation (Fig. 2G). Our IHC analyses of Runx2 expression in the tibial growth plate, furthermore, also corroborated Runx2 up-regulation in hypertrophic chondrocytes (Supplemental Fig. 3B, Table 2) and suggest a potential role for Runx2 in the regulation of ST2 splice variant expression.

### Runx2 activates ST2 and Runx3 expression in ATDC5 chondrocytes

**Figure 3:**
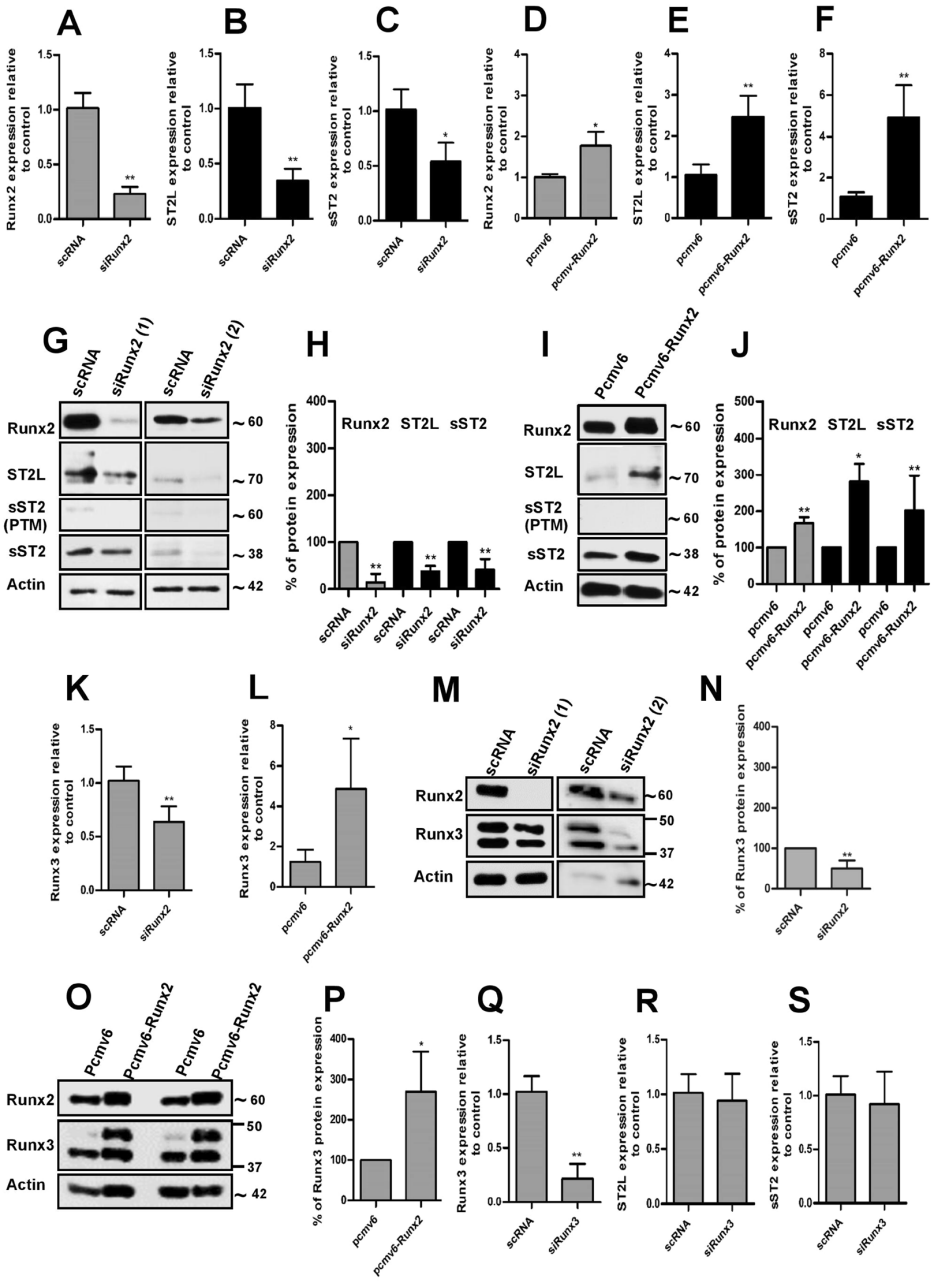
Runx2 regulates ST2 and Runx3 expressions in ATDC5. The evaluation of mRNA expression level of Runx2 (**A**), ST2L (**B**) and sST2 (**C**) in Runx2 silenced ATDC5 or Runx2 (**D**), ST2L (**E**) and sST2 (**F**) in Runx2-overexpressing ATDC5 cells (Runx2 cDNA stable transfection) by q-PCR (n = 5-6). (**G** and **H**) Representative immunoblotting and quantification of Runx2, ST2L and sST2 protein expressions in ATDC5 chondrocytes silenced by two different Runx2 siRNA sets. Transfection period was 72 hrs. (**I** and **J**) Representative immunoblotting and quantification of Runx2, ST2L and sST2 protein expressions in ATDC5 chondrocytes stably transfected with empty vector (pCMV6) or mouse Runx2 cDNA vector (pCMV6-Runx2) (n = 5-6). Figure I is the lane 1 and lane 4 of an immunoblot and full blot is shown in Supplemental Fig. 8. The qPCR evaluation of Runx3 mRNA level in siRunx2 ATDC5 (**K**) or Runx2 cDNA transfected (pCMV6-Runx2) ATDC5 cells (**L**) (n = 5-6). (**M**–**P**) The Runx3 protein expression analysis (representative immunoblotting and quantification) in Runx2 silenced (**M** and **N**) and Runx2-overexpressing ATDC5 chondrocytes (**O**–**P**). Runx2 knockdown was performed by two different Runx2 siRNA sets. Analysis was performed 48 to 72 hrs after siRNA or cDNA transfection. (**Q**–**S**) The qPCR of Runx3, ST2L and sST2 expressions in Runx3 silenced cells. The expression level of each gene was normalized to GAPDH. The protein quantifications are relative to their control and normalized to the respective β-actin level. *P ≤ 0.05, **P < 0.01.

Regulation of ST2 expression by Runx2 was achieved by transient knockdown or stable overexpression of Runx2 in undifferentiated and differentiating ADTC5 cells. Runx2 down-regulation resulted in the suppression of both ST2L and sST2 mRNA expression as compared to scrambled RNA transfected control cells. (Fig. 3A,B and C and Supplemental Fig. 4A,B). ST2L and sST2 mRNA expression levels were, accordingly, substantially induced in ATDC5 chondrocytes stably transfected (S.T) with mouse Runx2 cDNA (pcmv6-Runx2) as compared to empty vector transfected control cells (pcmv6), indicating a potential regulation of ST2 by Runx2. (Fig. 3D–F). In order to analyze the regulation of ST2 expression by Runx2 at the protein level, the molecular weights of ST2L and sST2 expressed in ATDC5 cells were primarily assessed. Whilst the predicted molecular weights of the ST2L and sST2 splice variants are 63 and 37 kDa, respectively, ST2 isoforms have, however, been shown to undergo post-translational modifications, in particular, glycosylation, resulting in different molecular weights^40,41^. The actual molecular weights of ST2L and sST2 expressed in ADTC5 cells were established by transiently transfecting these cells with murine ST2L and sST2 cDNA vectors containing DDK tag sequences (pcmv6-ST2L and pcmv6-sST2). Anti-DDK tag antibody detected two bands with molecular weights of approximately 70 and 85 kDa (Supplemental Fig. 5, black arrows) in cells ectopically expressing ST2L cDNA, and two bands with molecular weights of approximately 38 and 60 kDa in cells ectopically expressing sST2 cDNA (Supplemental Fig. 5, gray arrows). As previously reported, the larger bands (85 and 60 kDa) corresponded, in each case, to post-translationally modified (PTM) transmembrane and soluble forms respectively^40,41^.

A pronounced siRunx2 induced down-regulation of Runx2 protein was verified by immunoblotting analysis and quantification of the protein expression level (Fig. 3G,H). Runx2 down-regulation was accompanied by decreased ST2L and sST2 protein expression. (Fig. 3G,H). Runx2-overexpression, conversely, increased ST2L and sST2 protein expression (Fig. 3I,J). It should, however, be noted that signals indicating post-translational modification of ST2 isoforms in untreated or treated ATDC5 cells were negligible or undetectable, indicating that ST2 isoforms are principally present in an unmodified form in ATDC5 cells. We furthermore detected down-regulation of Runx3, another transcription factor from the runt-related family, in Runx2-silenced ATDC5 cells (Fig. 3K). Runx2-overexpressing cells accordingly exhibited significant up-regulation of Runx3 mRNA level (Fig. 3L).These results were further corroborated at protein level indicating Runx3 to be a novel Runx2 target in ATDC5 chondrocytes (Fig. 3M–P). Runx3 knockdown did not, however, prominently impact the expression of the ST2 isoforms (Fig. 3Q–S), leading to the conclusion that Runx2, but not Runx3 regulates expression of ST2 in ATDC5 chondrocytes.

### Runx2 activates ST2 and Runx3 expression in primary human growth plate chondrocytes

**Figure 4:**
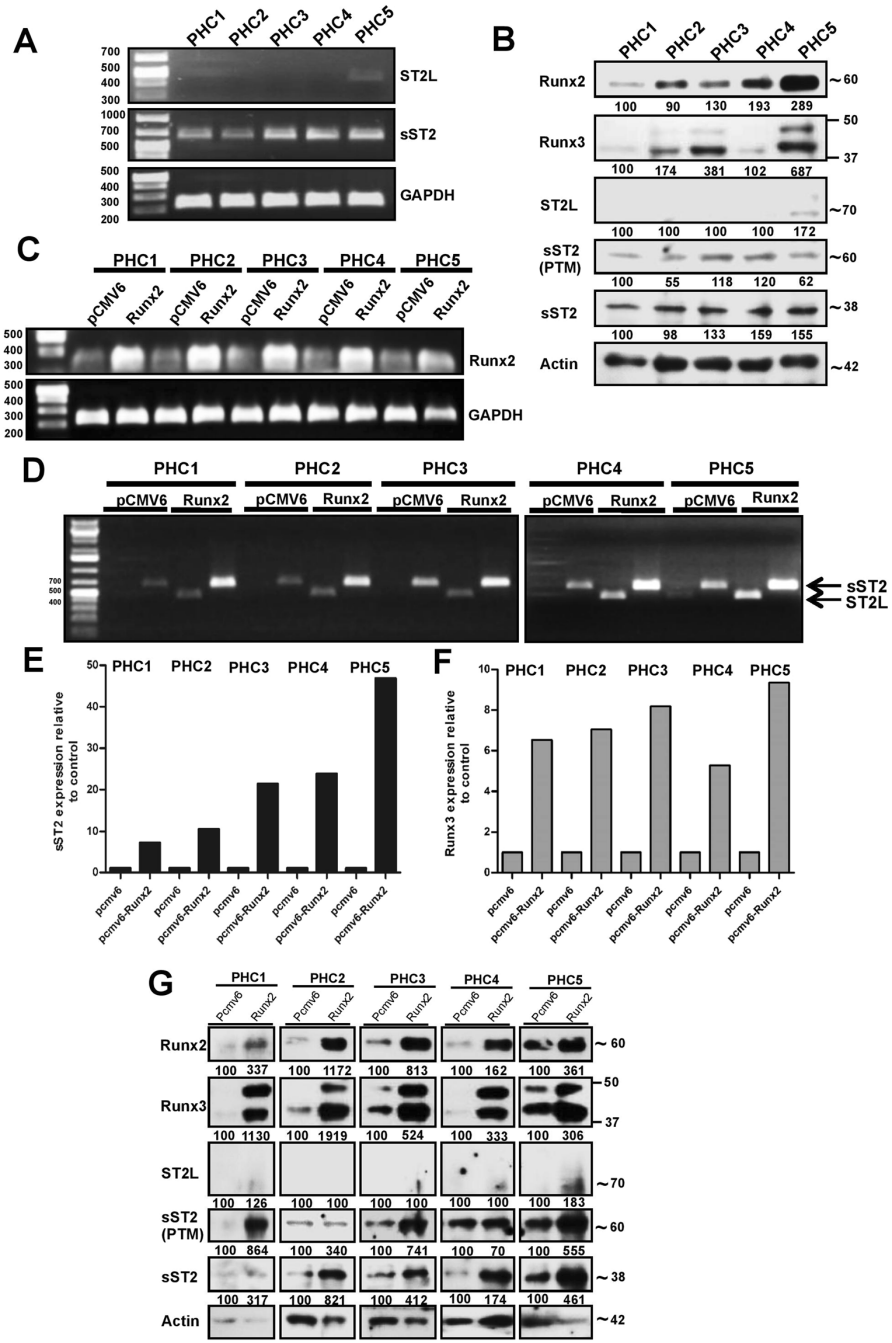
Runx2 regulates ST2 and Runx3 expression in primary human chondrocytes. (**A**) RT-PCR analysis of endogenous ST2L (454 bp) and sST2 (659 bp) mRNA expression in five PHCs (full gel is shown in Supplemental Fig. 6). The GAPDH was used as internal control. (**B**) The endogenous protein level of ST2L, sST2, Runx3, and Runx2 in five PHCs. (**C**) The RT-PCR of the expression of Runx2 in five PHCs transiently transfected with human Runx2 cDNA vector. (**D**) Primers specific to human ST2L and sST2 splice variants generating 454 and 659 bp PCR products respectively were used to analyze mRNA level 24 hrs after transient transfection of Runx2 cDNA vector. (**E** and **F**) The qPCR of the expression of sST2 and Runx3 in PHCs 24 hrs after transient transfection of Runx2 cDNA vector. (**G**) Immunoblotting of PHCs transiently transfected with empty vector (pCMV6) or human Runx2 cDNA vector (Runx2) as indicated by different antibodies. β-actin was utilized as a loading control. The numbers (% of protein expression) are relative to the control and normalized to the β-actin.

In order to examine the relevance of our findings to human samples, expression of ST2 splice variants and Runx3 was studied in PHCs. The chondrogenic phenotype of isolated PHCs was verified by the expression of the specific chondrocyte markers, Col II, aggrecan and Col X (Supplemental Fig. 7). Low level ST2L mRNA and protein expression were detected in only one PHC sample (PHC5), whereas sST2 mRNA and protein expression was detected in all five samples (Fig. 4A,B). It should be noted that signals indicating post-translational modification of sST2 were also detected in all PHC samples (Fig. 4B). Runx2 protein expression varied notably between the different samples, with the highest level in PHC5. A considerable correlation between Runx2 and sST2 isoform expression level could be detected in all PHCs. ST2L expression was however only detected in PHC5 when Runx2 expression levels were high (Fig. 4B). A correlation of Runx2-Runx3 expression was, furthermore, observed in samples PHC1, PHC3, and PHC5 (Fig. 4B). Transient overexpression of Runx2 in PHCs (Fig. 4C) was correlated with a strong induction of sST2 mRNA expression in all samples (Fig. 4D,E). Runx2 overexpression was, furthermore, associated with an induction of ST2L transcription in PHCs primarily lacking ST2L mRNA (Fig. 4D). Whilst Runx2 overexpression induced ST2L mRNA expression in all samples, an associated up-regulation of ST2L protein in these samples was undetectable or negligible in magnitude (Fig. 4G). Runx2 overexpression, however, resulted in a pronounced increase of unmodified sST2 isoform in all samples. A pronounced increase in signals indicating a PTM of the sST2 isoform was mainly observed in samples PHC1, PHC3, and PHC5 (Fig. 4G). Runx2 overexpression, moreover, strongly induced Runx3 expression in all PHC samples at both mRNA and protein levels (Fig. 4F,G), corroborating the results obtained in ATDC5 chondrocytes.

### ST2 splice variants are predominantly transcribed from the proximal promoter

**Figure 5:**
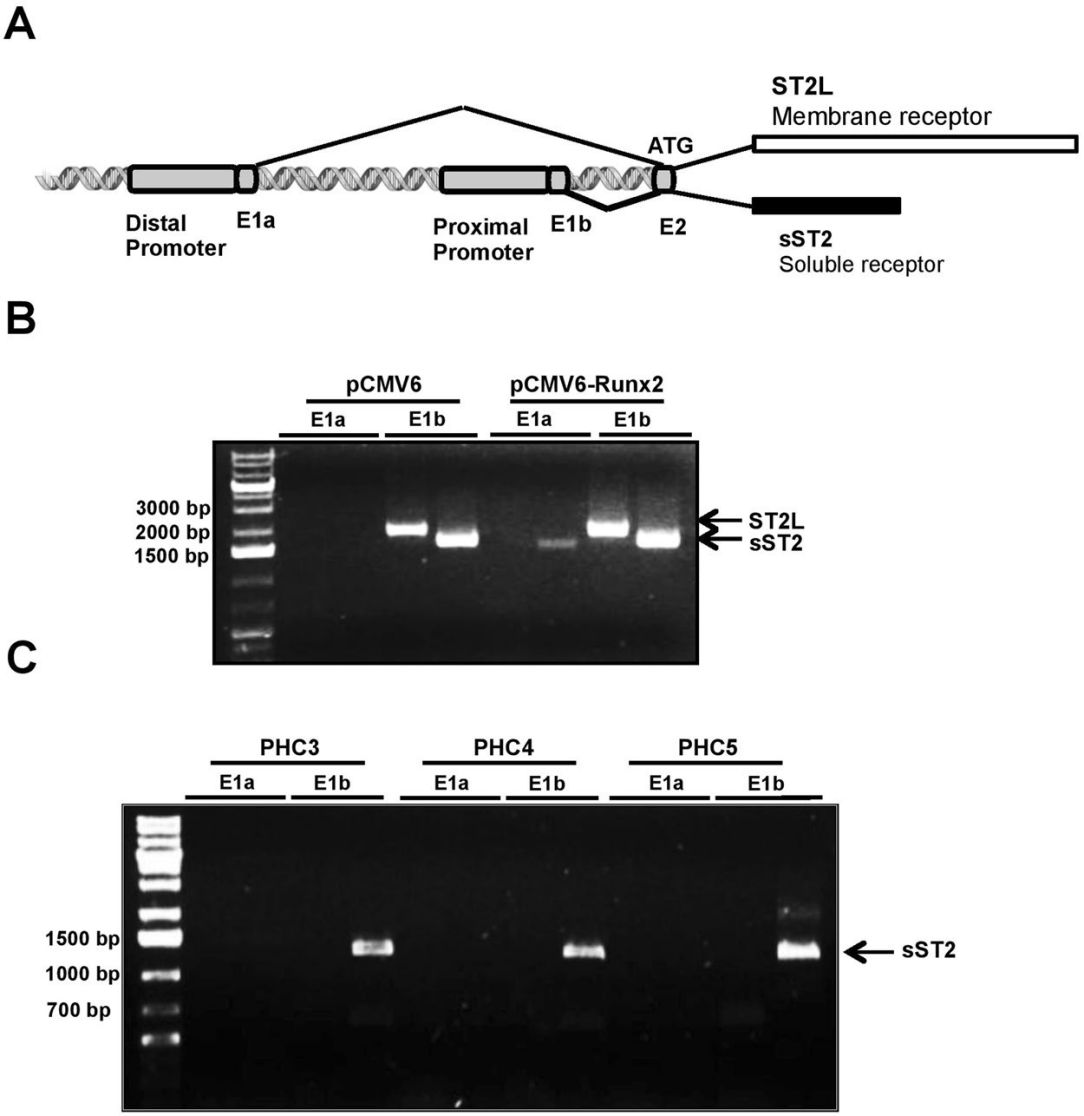
Distal and proximal promoter usage in ATDC5 and PHCs. (**A**) Schematic representation of 5′ promoter region of ST2 gene depicted up to exon2 (E2). Distal and proximal promoters are followed by non-coding E1a and E1b respectively. (**B**) Promoter usage analysis in ATDC5 chondrocytes. The ATDC5 stably transfected with empty vector (pCMV6) or mouse Runx2 cDNA vector (pCMV6-Runx2) were subjected to RT-PCR. The PCR product lengths representing ST2L and sST2 transcription from distal promoter are 1827 and 1718 bp respectively and transcription from proximal promoter generates 1863 bp fragment for ST2L and 1754 bp fragment for sST2^10^. (**C**) Promoter usage analysis exhibited E1b associated expression of 1237 bp fragment sST2 isoform in 3 different PHCs^8^.

The 5′ region of the ST2 gene structure up to exon 2 is depicted in Fig. 5A. Expression of ST2 isoforms are regulated by alternative, proximal and distal promoters which are located approximately 10.5 and 8 kb pairs distance from each other in the mouse and human ST2 genes, respectively^8,9^. Transcription initiation from either the distal or proximal promoter, starting from the concomitant non-coding exon 1a (E1a) or exon 1b (E1b) respectively, was shown to be cell type-specific^8–11^. Translation, however, starts from common exon 2 (E2) for transcripts initiated from either of distal or proximal promoters^10^. To examine which promoter drives ST2 expression in ATDC5 chondrocytes, RT-PCR was performed using primers annealing specifically to the murine E1a or E1b according to Hayakawa M. *et al*.^10^. Our results confirmed that transcription of both ST2L and sST2 initiated from the proximal promoter in control ATDC5 cells and following Runx2 overexpression (Fig. 5B). It should be noted that Runx2 overexpression also resulted in the transcription of sST2 from the distal promoter, indicated by an E1a primers-associated signal (Fig. 5B). The promoter usage primers indicated that PHCs also express sST2 from the proximal promoter (Fig. 5C). Taken together these results suggest that the proximal promoter is the dominant promoter for the initiation of ST2 isoform transcription.

### ST2 knockout mice exhibit a reduced hypertrophic zone in femur and tibial growth plates

**Figure 6:**
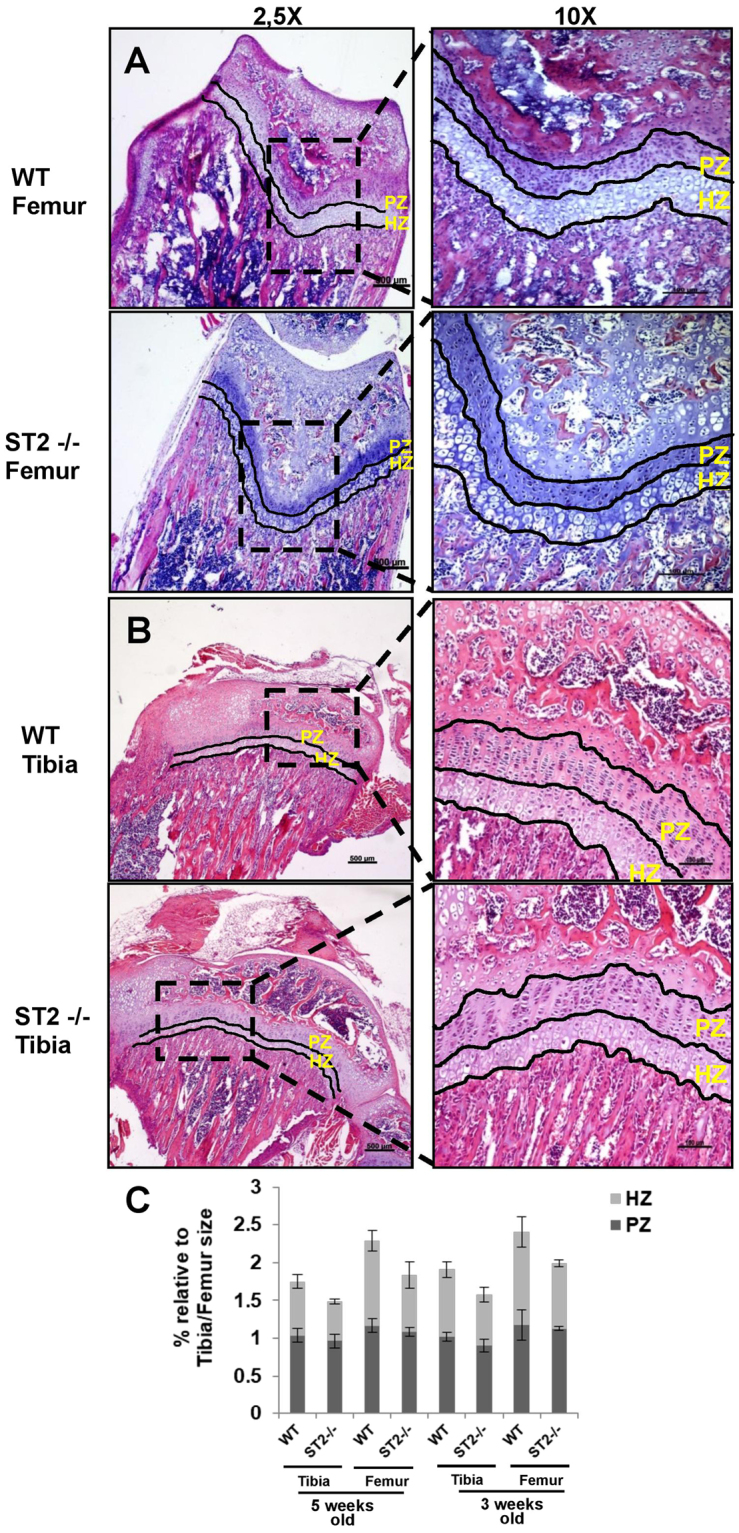
Hypertrophic zone reduction in ST2−/− mice. The H&E staining of femur (**A**) and tibial bones (**B**) of 5 week old WT (upper panels, left and right) and ST2−/− (lower panels, left and right) mice. The magnifications are 2,5x with scale bar of 500 µm and 10x with scale bar of 100 µm. (**C**) The graphs represent % of femur and tibial proliferative and hypertrophic zones relative to the respective femur/tibia full length in WT and ST2−/− mice at the age of 3 and 5 weeks old. For the size measurement, several randomly selected fields from 3 WT (6 tibias and 6 femurs) and 3 ST2−/− (5 tibias and 5 femurs) mice were analyzed. PZ, proliferative zone and HZ, hypertrophic zone.

**Figure 7:**
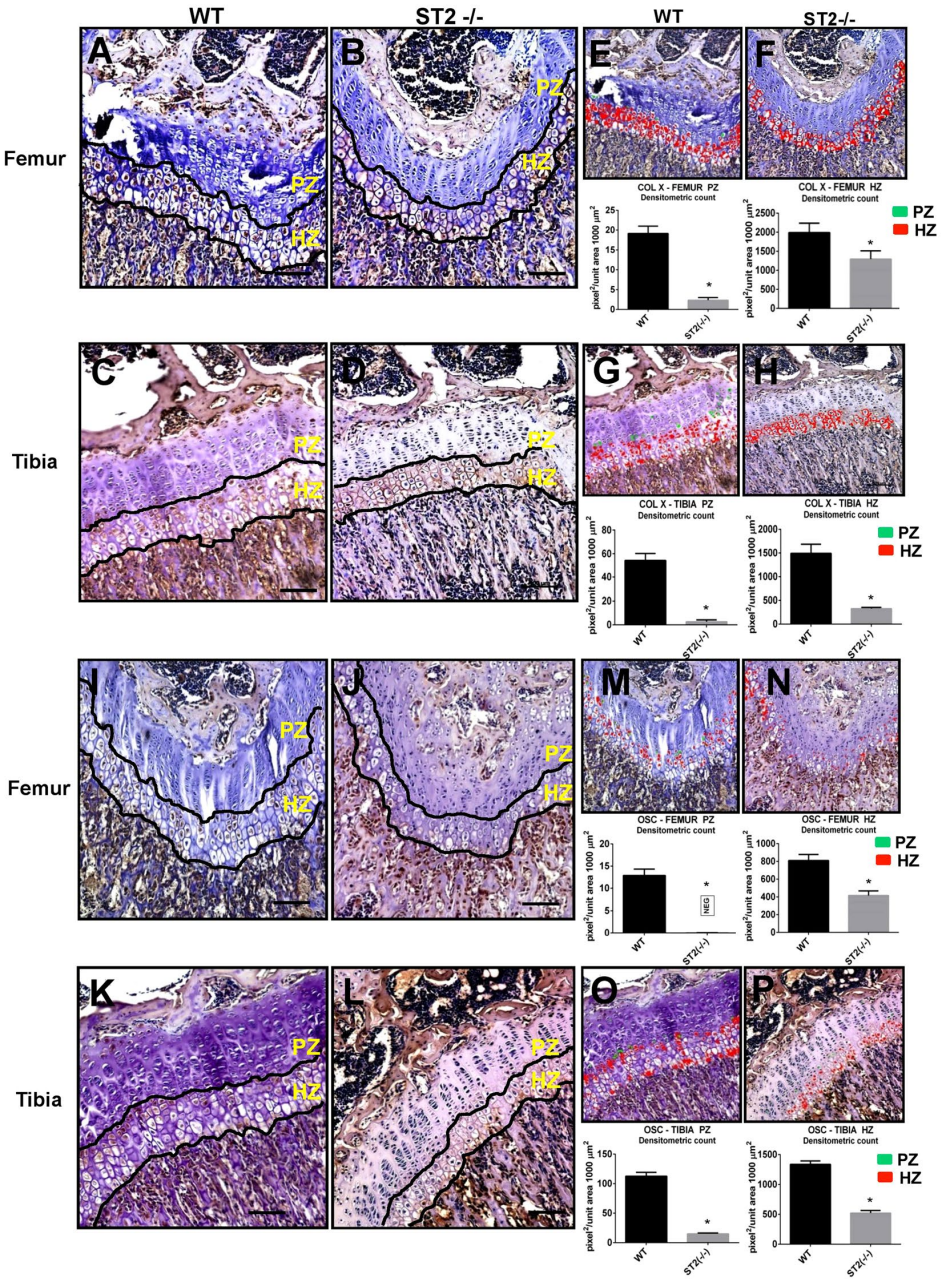
Decreased expression of Col X and OSC in ST2−/− mice. IHC analysis of Col X expression in femur of WT (**A**) and ST2−/− (**B**) mice and tibia of WT (**C**) and ST2−/− (**D**) mice. Densitometric analysis (pixel^2^/unit area 1000 µm^2^) of Col X in femur of WT (**E**) and ST2−/− (**F**) mice and tibia of WT (**G**) and ST2−/− (**H**) mice. IHC analysis of OSC expression in femur of WT (**I**) and ST2−/− (**J**) mice and tibia of WT (**K**) and ST2−/− (**L**) mice. Densitometric analysis (pixel^2^/unit area 1000 µm^2^) of OSC in femur of WT (**M**) and ST2−/− (**N**) mice and tibia of WT (**O**) and ST2−/− (**P**) mice. PZ, proliferative zone and HZ, hypertrophic zone. *P ≤ 0.05.

To evaluate the possible functional relevance of ST2 expression in chondrocytes, we analyzed femur and tibial growth plates isolated from 3 and 5 weeks old ST2−/− and WT mice by H & E staining. There was no considerable effect on the PZ of ST2−/− growth plates as compared to WT controls (Fig. 6A–C). The HZ of growth plates was, however noticeably reduced in the femur and tibia of ST2−/− mice, as indicated by size measurements of several different regions represented in % relative to the full femur/tibia lengths (Fig. 6A–C). The effect of ST2 knockout on the expression level of hypertrophic markers in the growth plates was subsequently analyzed. IHC and densitometric analysis of the hypertrophic and terminal differentiation markers Col X and OSC^42^ revealed marked decreases throughout the HZ of the growth plates in ST2−/− mice (Fig. 7A–P), suggesting likely a functional role for ST2 in controlling the expression level of hypertrophic markers *in vivo*.

### ST2 and Runx3 regulate expression of proliferative and hypertrophic markers in ATDC5

**Figure 8:**
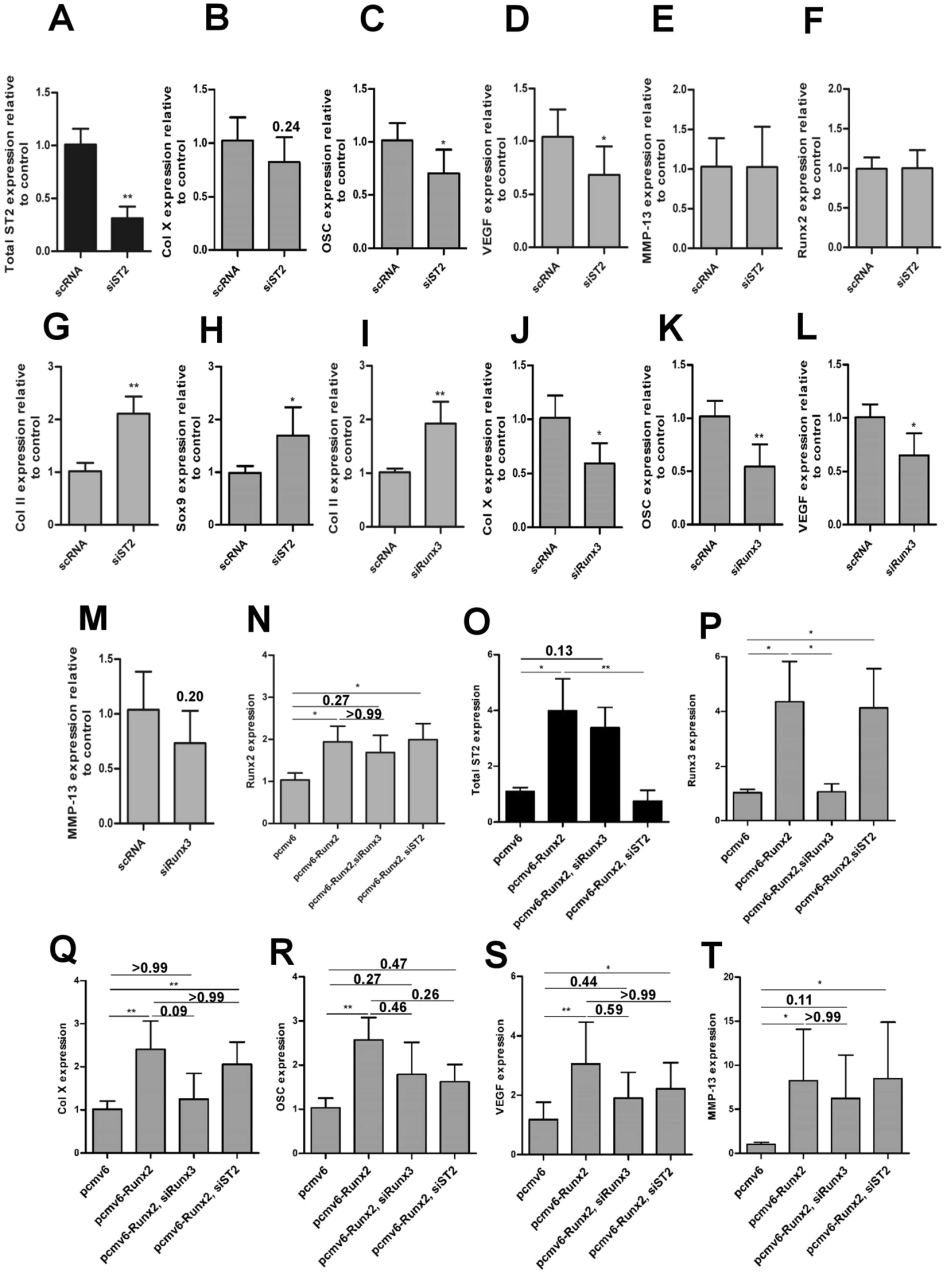
ST2 and Runx3 regulate expression of proliferative and hypertrophic markers in ATDC5. The qPCR evaluation of total ST2 (**A**), Col X (**B**), OSC (**C**), VEGF (**D**), MMP-13 (**E**), Runx2 (**F**), Col II (**G**) and Sox9 (**H**) in siST2 or Col II (**I**), Col X (**J**), OSC (**K**), VEGF (**L**) and MMP-13 (**M**) in siRunx3 ATDC5 cells. The qPCR analysis of Runx2 (**N**), total ST2 (**O**), Runx3 (**P**), Col X (**Q**), OSC (**R**), VEGF (**S**) and MMP-13 (**T**) in Runx2 overexpressing (pcmv6-Runx2), Runx2 overexpressing-Runx3 silenced (pcmv6-Runx2, siRunx3) and Runx2 overexpressing-ST2 silenced (pcmv6-Runx2, siST2) ATDC5 cells. The expression level of each gene was normalized to GAPDH and represented as the mean ± S.D. n = 5-6. (VEGF experiments: n = 7-8). *P ≤ 0.05, **P < 0.01.

siRNA-mediated ST2 knockdown, in ATDC5, resulted in a slight decrease in Col X expression. An ST2 silencing associated decrease in OSC expression was, in addition, pronounced, confirming the IHC observations from ST2−/− mice (Fig. 8A–C). The terminal hypertrophic marker VEGF was also repressed by the ST2 knockdown whilst MMP-13 and Runx2 were not influenced in ATDC5 cells (Fig. 8D–F). ST2 silencing was, moreover, associated with significant increases in expression of the proliferative stage markers Col II and Sox9 (Fig. 8G,H), and Runx3 silencing augmented Col II whilst leading to a repression of Col X, OSC, VEGF and MMP-13 expression in ATDC5 (Figs 3Q and 8I–M). These results suggest an involvement of ST2 and Runx3 in the regulation of ATDC5 chondrocyte differentiation, opposing proliferative and promoting hypertrophic differentiation. Runx2 and ST2 or Runx2 and Runx3 cooperative regulation of chondrocyte hypertrophy was, furthermore, established by separate ST2 and Runx3 knockdown in Runx2-overexpressing ATDC5 cells (Fig. 8N–T). The enhanced expression of Runx2, Runx3, total ST2, Col X, OSC, VEGF and MMP-13 was observed in ATDC5 cells stably transfected with a Runx2 cDNA vector (Fig. 8N–T). ST2 knockdown in these cells moderately decreased expression of Col X (Fig. 8O,Q). The OSC and VEGF expressions but not MMP-13 was also reduced by the ST2 knockdown in these cells (Fig. 8R–T). Runx3 silencing, however, repressed Col X expression to approximately basal level and led to a reduction of OSC, VEGF and MMP-13 expression (Fig. 8P–T). Furthermore, whilst ST2 silencing did not change the mRNA expression of Runx2, Runx3 knockdown slightly decreased it (Fig. 8N). These findings indicate that Runx2-associated activation of ST2 and Runx3 mediates induction of hypertrophic markers Col X, OSC, VEGF and MMP-13 leading to the cooperative regulation of hypertrophic differentiation in ATDC5 chondrocytes.

## Discussion

Whilst no, or only low-level ST2 expression was observed in the growth plate proliferative zone, strong cytoplasmic ST2 staining was detected in the pre-hypertrophic and hypertrophic zones. This result is consistent with the prominent cytoplasmic staining of sST2 in the hypertrophic chondrocyte zone during *in vitro* cultivation of mandibular condyle reported by Werenskiold *et al*.^12^. ST2L and sST2 also progressively increased during the hypertrophic phase in differentiating ATDC5. Prominent expression of sST2, and to a lesser extent of ST2L (in one of five human samples), has also been verified in primary human growth plate chondrocytes. sST2 has, in contrast, been discussed as an early differentiation marker during osteoblastogenesis, since sST2 expression coincides with the expression of the early osteogenic markers, col I and osteonectin and precedes expression of alkaline phosphatase and osteocalcin, markers of late osteoblastogenesis in MC3T3-E1 and KM-1K cells. ST2L expression, however, was below the detection limit^12^. Furthermore, whilst ST2L is expressed in mouse calvarial osteoblasts, this expression is reduced significantly in the course of differentiation^13^. The differences in the time-dependent expression patterns of ST2 isoforms during osteogenic and chondrogenic differentiation, therefore, indicate a different differentiation-dependent role in these tissues.

ST2 upregulation during late stages of ATDC5 differentiation was accompanied by enhanced expression of hypertrophic markers Runx2, Col X and MMP-13 (Col X and MMP-13 are known Runx2 target genes). Enhanced Runx2 expression during chondrogenic differentiation of ATDC5 has been previously documented in a number of publications^28,43–48^. Runx2 activation is, furthermore, initiated during the pre-hypertrophic phase and maintained throughout the hypertrophic stage *in vivo* ^17,18,39^. Runx2 induces chondrocyte hypertrophy by binding to and activating the promoters of target genes such as Col X^38^. Silencing of Runx2 expression by siRNA resulted in the interference with ST2L and sST2 mRNA and protein expression in ATDC5. Consistent with this, activation of ST2 isoforms by Runx2 was observed after stable transfection of ATDC5 cells with a Runx2 cDNA. Transient overexpression of Runx2 in PHCs, induced expression of sST2 transcript and protein. Whilst ST2L mRNA expression was induced following Runx2 transfection, a slight increase in the level of ST2L protein was observed. It should, however, be noted that ST2L protein expression was detected in a PHC sample with high endogenous Runx2 levels. We thereby observed a distinct Runx2-associated regulation of sST2 expression and ST2L mRNA expression in both cell systems. The minor discrepancies between ST2 and Runx3 mRNA levels with their corresponding protein expression levels in ATDC5 and PHCs post silencing or post Runx2 cDNA transfection might be due to the methodological differences. The absence or low ST2L protein in PHCs might have different causes. Runx2-induced ST2L mRNA expression was relatively weak compared to Runx2-induced sST2 mRNA levels in PHCs. The ST2L protein level might, therefore, have been below the detection limit for immunoblotting. Differentiation-dependent expression of ST2L should, furthermore, also be considered, since the highest expression level of ST2L associated with the Runx2 level was observed in the hypertrophic stage of chondrocyte differentiation. The association of Polydactyly with different autosomal-dominant mutations in genes or in cis-regulatory elements in the Hoxa- or Hoxd clusters, the Wnt signalling pathway or Notch^49^ should also be taken into consideration. Discrepancies in Runx2 associated ST2L protein levels in ATDC5 and PHCs might, therefore, also be due to genetic differences in these cell systems. Distinct Runx2-induced expression of ST2L transcripts and sST2 transcripts and proteins was, however, demonstrated in both cell systems, indicating ST2 to be a new Runx2 target gene in the hypertrophic stage of chondrocyte differentiation.

Runx2 and Runx3 *in situ* hybridization indicated co-expression of these transcription factors in developing cartilage in mice embryos^50^. Runx3 expression is induced in pre-hypertrophic zones, maintained during hypertrophy and declines during terminal chondrocyte hypertrophy^51^, resembling the pattern of Runx2 expression during different stages of chondrogenic differentiation. In line with these studies, Runx3 was found to be regulated by Runx2 in our cell systems, indicating Runx3 to be another new Runx2 target in chondrocytes. The Runx3 gene contains two promoters designated P1 and P2. P1 harbors 2 adjacent Runx binding sites adjacent to the transcription start site which are conserved between mouse and human^52^. Direct Runx3 activation involving a binding of Runx2 to the Runx3 promoter is, therefore, conceivable. Runx3 knockdown, however, did not change the expression level of ST2, thus indicating that Runx2 is the main inducer of ST2 in chondrocytes.

Cell-type specific regulation of ST2 gene by distal and proximal promoters has been shown in multiple studies^8–11^. In murine and human fibroblasts, transcription of both soluble and transmembrane ST2 initiates at the proximal promoter, whilst in murine mast cells or thymoma cell line EL-4, ST2 transcription is initiated at the distal promoter^8–10^. In the human leukaemic cell line UT-7, the distal promoter is dominantly activated for the expression of both ST2 isoforms, whilst the ST2 mRNA can, to some extent, also be transcribed from the proximal promoter^8^. In the FIT-1 gene (the rat homologue of ST2), ST2L is transcribed from the distal promoter, whilst the soluble form is generated mainly from the proximal promoter, showing a promoter-specific production of ST2 isoforms^11^. In both ATDC5 chondrocytes and PHCs, ST2 isoforms are transcribed from the proximal promoter, even though the distal promoter is also capable of producing sST2 transcripts in ATDC5 cells. Thus, it can be concluded that whilst the proximal promoter is the dominant promoter responsible for transcribing both isoforms, sST2 can also be expressed from the distal promoter.

The functional relevance of ST2 was assessed by comparing femur and tibial growth plates of ST2−/− mice with WT controls. Although ST2−/− PZ morphology seemed to be comparable to WT mice, the size of the HZ in ST2−/− mice was apparently reduced, implying likely a ST2 function during chondrocyte hypertrophy *in vivo*. Hypertrophic zone reduction was accompanied by lower expression of the hypertrophic markers Col X and OSC, suggesting that ST2 probably modulates chondrocyte hypertrophy *in vivo*. The mild but perceptible reduction in hypertrophy observed in ST2−/− mice, however, indicates that ST2 likely plays a contributive role in this stage. The observed regulatory influence of ST2 on hypertrophic differentiation was further substantiated by *in vitro* testing of siRNA-mediated ST2 suppression in ATDC5 chondrocytes. ST2 suppression repressed OSC and VEGF and slightly diminished Col X, but enhanced Sox9 and Col II, revealing positive and negative impacts on hypertrophic and proliferative stage markers respectively. The slight discrepancy between the siST2 effect on Col X expression in ATDC5 with that observed in ST2−/− mice can most likely be ascribed to the more prominent effect of ST2 induced by full ablation in knockout mice. Since ST2 does not seem to affect Runx2 mRNA expression as shown in our study and by Werenskiold, A *et al*.^14^, there is a need for further elucidation of the molecular mechanisms underlying ST2 dependent regulation of differentiation markers. A potential upstream mechanism might comprise IL-33 binding to ST2L, thereby stimulating MAPK and NFkB^53^, two major signaling pathways involved in chondrocyte proliferation, differentiation and cell death^54–56^. ST2L-associated regulation of osteogenic differentiation has been previously shown in osteosarcoma cells^14^ and analysis of the skeletal phenotype has indicated enhanced bone resorption in ST2 deficient mice highlighting a negative influence of IL-33/ST2 signaling pathway on the osteoclastogenesis that was corroborated by *in vitro* studies as well^13^. Our study thus provides the first indication of a role for ST2 in the regulation of chondrogenic differentiation.

This study also demonstrated Runx3 to be another Runx2 target in chondrocytes. Runx3 silencing suppressed Col X, OSC and VEGF whilst inducing Col II expression, showing that Runx3 functions in a pattern similar to that observed for ST2. Our results support a previous study showing that gain and loss of Runx3 function modulates the early and late differentiation markers Col II and Col X in the limb bud-derived cell line MLB13MYC^57^. Runx3 expression is induced and sustained during chondrocyte hypertrophy, and in Runx3-knockout mice embryos, chondrocyte hypertrophy and vascular invasion into cartilage are slightly delayed^51^. Maturational blockage of chondrocytes in Runx2−/− mice was, furthermore, observed to a lesser extent than in Runx2−/−Runx3−/− mice completely lacking chondrocyte maturation^51^. These reports showcased a function for Runx3 during the hypertrophic stage of chondrocyte differentiation. In parallel to these ST2 and Runx3 investigations, silencing of either ST2 or Runx3 in Runx2-overexpressing cells resulted in differential down-regulation of Col X. Whereas Runx3 knockdown remarkably suppressed Runx2-associated upregulation of Col X, ST2 knockdown only moderately reduced it. ST2 and Runx3 silencing, however, reduced Runx2-mediated OSC and VEGF induction to a more or less similar extent, revealing a contribution by these two factors to chondrocyte hypertrophy and terminal differentiation. Furthermore, MMP-13 expression was decreased by Runx3 knockdown in ATDC5 or Runx2 transfected ATDC5 cells whilst ST2 silencing did not alter MMP-13 expression suggesting Runx3 but not ST2 to be involved in the control of MMP-13 expression during late hypertrophic differentiation. The ST2, therefore, seems to selectively target genes associated to the chondrocytes hypertrophic development in ATDC5 cells. Given also that Runx2 is slightly reduced in Runx3 silenced cells, it might thus contribute moderately to the further downregulation of target genes in our *in vitro* experimental system. This effect, however, could be additive considering the *in vivo* milder impact of Runx3−/− on the hypertrophic phenotype as compared to that induced by Runx2−/−^51^. It should be pointed out that although this study suggests a functional role for ST2 *in vivo*, further experimental examinations of growth plate development in ST2−/− mice in particular, prenatally are required to further corroborate the ST2 dependent regulation of hypertrophic differentiation *in vivo*. Overall, the principal conclusion of this study is that ST2 and Runx3 play a major role in the regulation of hypertrophic differentiation in ATDC5 chondrocytes as two novel targets of the key transcription factor Runx2.

**Figure 9:**
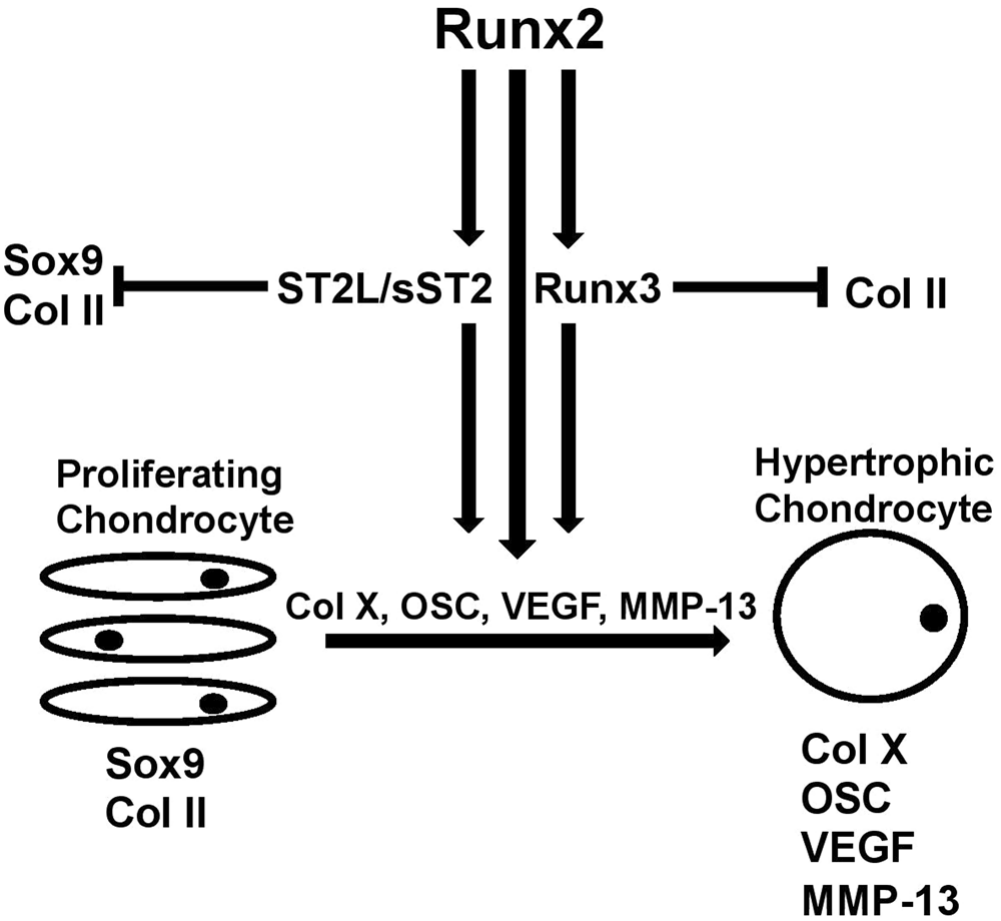
Proposed model for the contributing effect of Runx2 downstream targets ST2 and Runx3 in promoting chondrocyte hypertrophy in ATDC5 chondrocytes. In our suggested model, Runx2 induces expression of chondrocyte differentiation regulators ST2 and Runx3. These differentiation regulators promote ATDC5 chondrocyte hypertrophy through inhibition of proliferative stage markers Sox9 and Col II and induction of hypertrophic stage markers Col X, OSC, VEGF, and MMP-13.

Figure 9 depicts a proposed model for the promotion of ATDC5 hypertrophy through Runx2-dependent activation of the novel differentiation regulator ST2 and Runx3.

## Electronic supplementary material

Supplemental Figure
