# Supplementary material for: Runx2 mediated Induction of Novel Targets ST2 and Runx3 Leads to Cooperative Regulation of Hypertrophic Differentiation in ATDC5 Chondrocytes
Source: Sci Rep. 2017 Dec 20;7:17947. doi: 10.1038/s41598-017-18044-z (PMC5738421; doi:10.1038/s41598-017-18044-z)
Supplement: Supplementary file 1 — Supplemental Figure [file 41598_2017_18044_MOESM1_ESM.pdf]

Runx2 mediated Induction of Novel Targets ST2 and Runx3 Leads to Cooperative Regulation of Hypertrophic Differentiation in ATDC5 Chondrocytes

Ehsan Bonyadi Rad[1*], Giuseppe Musumeci[2] , Karin Pichler[3,4], Maryam Heidary[5], Marta Anna Szychlinska[2] , Paola Castrogiovanni[2], Egon Marth[6], Christina Böhm[7], Sriveena Srinivasaiah[1], Gerhard Krönke[7], Annelie Weinberg[1] & Ute Schäfer[8]

[1] Department of Orthopedics and Trauma Surgery, Medical University Graz, Austria;

[2] Department of Biomedical and Biotechnological Sciences, Human Anatomy and Histology Section, School of Medicine, University of Catania, Italy;

[3] Department of Children and Adolescent Medicine, Pediatrics I, Medical University of Innsbruck, Austria;

[4] Division of Neonatology, Pediatric Intensive Care and Neuropediatrics, Department of Pediatrics, Medical University of Vienna, Vienna, Austria;

[5] Translational Research Department, Institute Curie, Paris, France;

[6] Institute of Hygiene, Microbiology and Environmental Medicine, Medical University Graz, Austria;

[7]Friedrich-Alexander-University Erlangen-Nürnberg (FAU), Department of Internal Medicine 3 – Rheumatology and Immunology, Universitätsklinikum Erlangen, Erlangen, Germany.

[8] Department of Neurosurgery, Medical University Graz, Austria;

[*] **Corresponding author**

Ehsan Bonyadi Rad (ehsan.bonyadirad@medunigraz.at.)

Supplementary Figures

Fig. 1

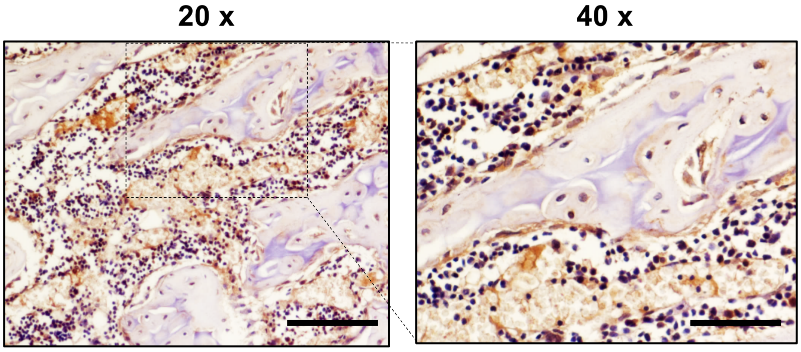

Fig. 2

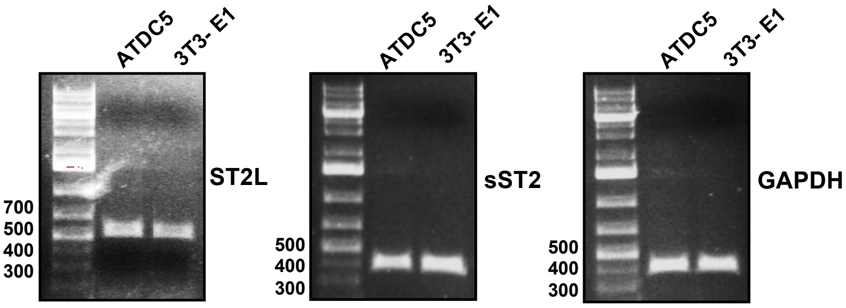

Fig. 3 A

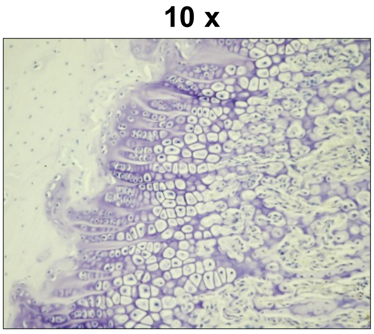

Fig. 3 B

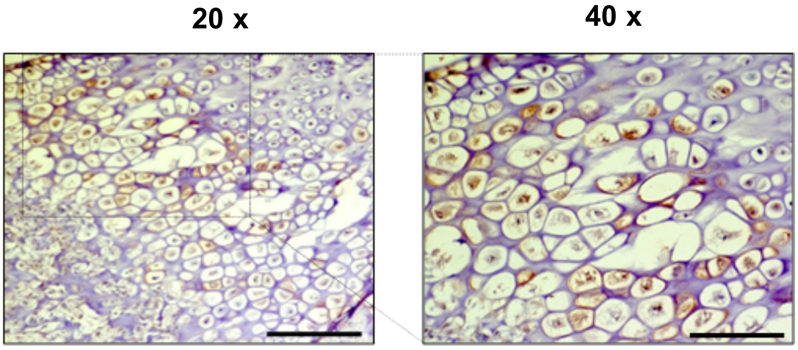

Fig. 4 A

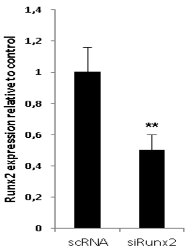

Fig. 4 B

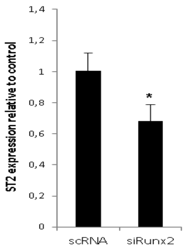

Fig. 5

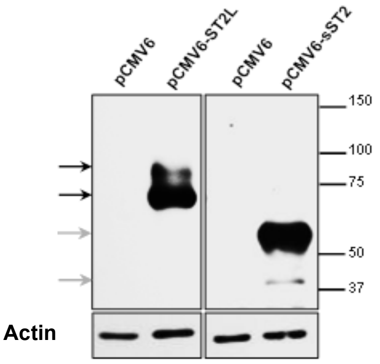

Fig. 6

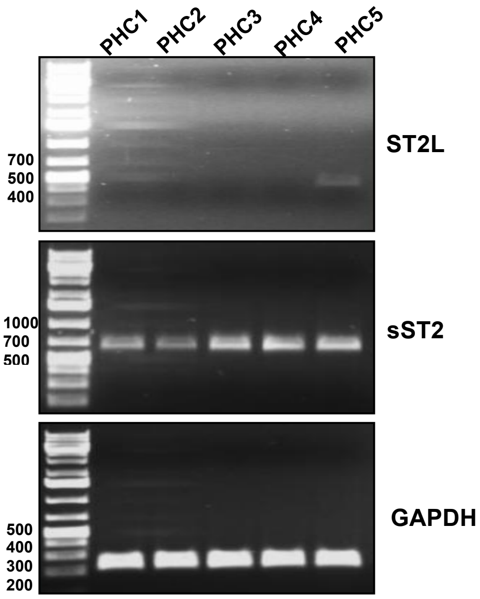

Fig. 7

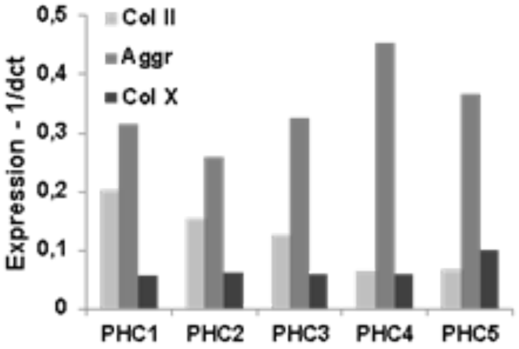

Fig. 8

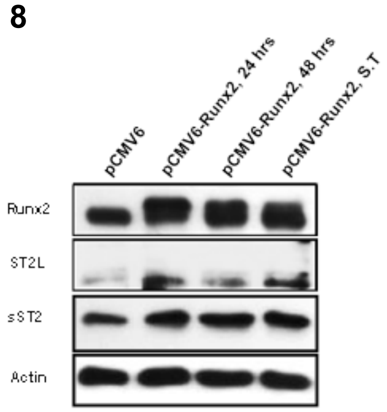

Supplemental Figure Legends

Supplemental figure 1. Immunohistochemical staining of ST2 in the trabecular bone of tibia of three week old mice. Magnification of 20x and 40x are shown. Scale bar is 100µm for 20x and 50µm for 40x pictures.

Supplemental figure 2. Full gels of the endogenous expression of ST2L, sST2 and GAPDH in ATDC5 and MC3T3- E1 cells.

Supplemental figure 3. (A) Negative control staining, in tibial growth plate. (B) Immunohistochemical staining of Runx2 in the tibial growth plate at the age of three weeks. Magnification of 20x and 40x are shown. Scale bar is 100µm for 20x and 50µm for 40x pictures.

Supplemental figure 4. Runx2 regulates ST2 expression in differentiating ATDC5. (A) Runx2 knockdown in differentiating ATDC5. (B) Expression of total ST2 in Runx2 silenced ATDC5 cells in a differentiation condition. Gene silencing analysis was performed 48 hrs post transfection.

Supplemental figure 5. Post transcriptional modification of ST2L and sST2 isoforms. Immunoblotting of ATDC5 protein lysates transiently transfected with pCMV6, murine ST2L (PCMV6-ST2L) or murine sST2 (PCMV6-sST2) cDNA vectors. Transfer to polyvinylidene difluoride membrane was performed and ST2L (black arrows) and sST2 (gray arrows) bands were detected by anti-DKK antibodies.

Supplemental figure 6. Full gels of the endogenous expression of ST2L, sST2 and GAPDH in 5 PHCs.

Supplemental figure 7. Chondrogenic phenotype of PHCs was assessed by the qPCR evaluation of Col II, Aggrecan and Col X expression.

Supplemental figure 8. Runx2 regulates ST2L and sST2 expression in ATDC5 chondrocytes. A full blot of the effect of forced Runx2 expression on ST2L and sST2 splice variants expression. ATDC5 cells were transiently (24 hrs, lane2 and 48 hrs, lane 3) or stably (S.T, lane 4) transfected with pCMV6 or pCMV6-Runx2 vectors. After transfer to polyvinylidene difluoride membrane, expression level of Runx2, ST2L, sST2 and β-actin were assessed by respective antibodies.

References of RT-PCR and qPCR primers used in this study.

1. Cho, H. J. *et al*. Vascular calcifying progenitor cells possess bidirectional differentiation potentials. *PLoS Biol.* **11**, e1001534 (2013).

2. Huang, Y. *et al*. Inhibition of b-catenin signaling in chondrocytes induces delayed fracture healing in mice. *Journal of Orthopaedic Research* **30**, 304-310 (2012).

3. Kobayashi, K. *et al*. Mitochondrial superoxide in osteocytes perturbs canalicular networks in the setting of age-related osteoporosis. *Sci. Rep.* **5**, 9148 (2015).

4. Tezuka, H. *et al*. Regulation of IgA production by naturally occurring TNF/iNOS-producing dendritic cells. *Nature* **448**, 929-933 (2007).

5. Crnkovic, S. *et al*. Functional and molecular factors associated with TAPSE in hypoxic pulmonary hypertension. *Am. J. Physiol. Lung Cell. Mol. Physiol.* **311**, L59-73 (2016).

6. Cheng, C. K. *et al*. Transcriptional repression of the RUNX3/AML2 gene by the t(8;21) and inv(16) fusion proteins in acute myeloid leukemia. *Blood* **112**, 3391-3402 (2008).

7. Mun, S. H. *et al*. Interleukin-33 stimulates formation of functional osteoclasts from human CD14(+) monocytes. *Cell Mol. Life Sci.* **67**, 3883-3892 (2010).

8. Li, Z. Y. *et al*. Epithelial Membrane Protein 1 Inhibits Human Spinal Chondrocyte Differentiation. The Anatomical Record: Advances in Integrative Anatomy and Evolutionary Biology 294, 1015-1024 (2011).

9. Suckow, A. T. *et al.* Transcellular Neuroligin-2 Interactions Enhance Insulin Secretion and Are Integral to Pancreatic ß Cell Function. *Journal of Biological Chemistry* **287**, 19816-19826 (2012).

10. Huang, C. *et al*. Dual-specificity histone demethylase KIAA1718 (KDM7A) regulates neural differentiation through FGF4. *Cell Res.* **20**, 154-165 (2010).

11. Hayakawa, M. *et al.* T-helper type 2 cell-specific expression of the ST2 gene is regulated by transcription factor GATA-3. *Biochim. Biophys. Acta* **1728**, 53-64 (2005).

12. Shen, L., Jin, Y., Freeman, G. J., Sharpe, A. H. & Dana, M. R. The Function of Donor versus Recipient Programmed Death-Ligand 1 in Corneal Allograft Survival. *The Journal of Immunology* **179**, 3672-3679 (2007).

13. Li, H. *et al*. The Cloning and Nucleotide Sequence of Human ST2L cDNA. *Genomics* **67**, 284-290 (2000).

14. Appel, S. *et al*. Epithelial-specific transcription factor ESE-3 is involved in the development of monocyte-derived DCs. *Blood* **107**, 3265-3270 (2006).
